# Concerns regarding proposed groundwater Earth system boundary

**DOI:** 10.1038/s41586-024-08082-9

**Published:** 2024-11-20

**Authors:** M. O. Cuthbert, T. Gleeson, M. F. P. Bierkens, G. Ferguson, R. G. Taylor

**Affiliations:** 1 School of Earth and Environmental Sciences, Cardiff University, Cardiff, UK; 2 Department of Civil Engineering and School of Earth and Ocean Science, University of Victoria, Victoria, British Columbia Canada; 3 Department of Physical Geography, Utrecht University, Utrecht, The Netherlands; 4 Deltares, Unit Soil and Groundwater Systems, Utrecht, The Netherlands; 5 Department of Civil, Geological and Environmental Engineering, University of Saskatchewan, Saskatoon, Saskatchewan Canada; 6 Global Institute for Water Security, University of Saskatchewan, Saskatoon, Saskatchewan Canada; 7 Hydrology and Atmospheric Sciences, University of Arizona, Tucson, AZ USA; 8 Department of Geography, University College London, London, UK

**Keywords:** Hydrology, Environmental impact

**arising from**: J. Rockström et al. *Nature* 10.1038/s41586-023-06083-8 (2023).

Groundwater, the largest store of accessible freshwater on Earth, is under increasing pressure as a resource^1^ and its over-exploitation can harm dependent ecosystems, increase the severity of hydrological drought, and cause land subsidence and salinization^2^. It is, therefore, a welcome development that groundwater has been explicitly incorporated in the endeavour to quantify safe and just Earth system boundaries (ESBs) in a recent article by Rockström et al.^3^. However, here we raise concerns about the proposed groundwater ESB, by showing how it is based on an incorrect conceptual understanding of groundwater system dynamics and nomenclature. The proposed boundary is thus potentially misleading, with important implications for assessing the global status of groundwater resources with regards to safe and just water management.

Groundwater systems exist in a state of dynamic response to their imposed boundary conditions such as variations in climate, land cover, sea level and pumping^4^. They respond over timescales from months and years up to thousands of years for the world’s most important large aquifers^5^. Hence, there are often long-timescale transients in large-scale groundwater storage (GWS), which may be greatly lagged and attenuated compared with the current state of the boundary conditions.

Following any new, sustained groundwater pumping, groundwater storage is always initially reduced to a greater or lesser extent. Gradually, again over long timescales for large aquifers^6^, the rate of storage loss slows until the rate of pumping is matched by a combination of decreases in flows of groundwater that would have naturally discharged to rivers, the sea or evaporating vegetation, and by changes (normally increases) in any replenishment that the system is receiving (for example, from rainfall or streamflow losses). This is the concept of ‘capture’ whereby the system may approach a new dynamic equilibrium^7^, unless the rate of pumping is such that the maximum rate of capture is exceeded^8^, in which case, ongoing GWS decreases will occur.

Crucially, what can be considered renewable groundwater use is dependent on the spatio-temporal distribution of pumping, and is not an inherent property of an aquifer^9^. What may be considered sustainable groundwater use may thus be a small fraction of the renewable use, owing to value-laden and culturally variable judgements about what is considered ‘safe’ and ‘just’^9^.

A methodology that combines Gravity Recovery and Climate Experiment (GRACE) satellite observations with global hydrological models was used to assess whether “the subglobal safe ESB is met for a given aquifer when local drawdown does not exceed average annual recharge”^3^. This amounts to a subglobal safe ESB requiring no net decrease in GWS during the reference period used (2003–2016). It is noted that, having deciphered the hydrologically incorrect nomenclature, we checked the published code^3^ and then clarified our correct understanding of the method with the authors via personal correspondence before submitting this Matters Arising. A correct subglobal ESB concept is critical as “the global ESB for groundwater is that the subglobal ESB is met for all aquifers around the world”^3^. Unfortunately, the understanding of groundwater dynamics we describe above reveals fundamental problems with the proposed subglobal safe groundwater ESB including:

1. Historic pumping may have already reached a new dynamic equilibrium before the reference period for which a subglobal ESB is calculated. If so, previous pumping could have been devastating ecosystems, causing subsidence and inducing saline intrusion, and still be ascertained as ‘safe’ according to the proposed definition.
2. Some decrease in GWS must occur when groundwater is pumped. Hence for a system that is initially in dynamic equilibrium over a similar timescale to the reference period, no new groundwater abstraction at all is possible under ‘safe’ conditions of the proposed subglobal ESB.
3. If there is a trend downwards (natural or otherwise) within the reference period, for reasons that have nothing to do with pumping, or the error in the GWS estimation implies an incorrect downwards trend, a subglobal ESB could be transgressed even without any groundwater being pumped.
4. If there is a trend upwards (natural or otherwise) within the reference period for reasons that have nothing to do with pumping, or the error in the GWS estimation causes an incorrect upwards trend, a subglobal ESB might not be transgressed despite sufficient pumping occurring to cause significant harm.

The proposed subglobal ESB will probably catch instances of over-abstraction in already documented ‘hotspots’^10^, where pumping exceeds the maximum capture leading to persistent GWS decline. However, because the approach does not rigorously account for natural variability in recharge and discharge dynamics^4^, or the timescales and mechanics of groundwater pumping hydraulics^5–9^, the method is not fit for purpose and results drawn from this method are potentially misleading and unsafe.

The proposed boundary is also not just, for at least two reasons. First, some groundwater storage depletion is inherently necessary to extract groundwater^7^, meaning that maintaining a ‘safe’ subglobal ESB excludes some of the poorest people in the world and their future generations, who might have access to unused aquifers, from beginning to abstract any groundwater to improve their livelihoods. For example, climate-resilient development pathways identified by the Government of Niger in one of the poorest, yet rapidly growing, regions on the planet (Maradi, Niger) are contingent on groundwater withdrawals from an undeveloped sandstone aquifer^11^.

Second, under the proposed boundary, many already over-abstracted aquifers, from which wealthier nations have already benefited (for example, via irrigated or industrial productivity before the GRACE reference period), may still be within a subglobal groundwater ESB, for instance, if long-term pumping has led to a new equilibrium with overall lower groundwater levels. The paper^3^ argues that interspecies justice and future intergenerational justice are not met if local GWS declines over time, but without framing the boundary robustly within an appreciation of the groundwater system dynamics, this is potentially increasing environmental injustice instead.

Methodologically, insufficient attention has been paid to uncertainties in the calculation of changes in GWS from GRACE satellite data, which requires the deduction of highly uncertain estimates of storage changes for other terrestrial water stores from global-scale models^12^. By analysing at 0.25° resolution for the subglobal ESB, the authors^3^ also overlooked the explicit resolution guidance that comes with the published RL06.2 dataset they have used (https://www2.csr.utexas.edu/grace/RL06_mascons.html) which has a native resolution as per the CSR RL06 mascon solutions of 1°, and says “users must exercise caution when using these solutions in basins smaller than approximately 200,000 km^2^. Moreover, these solutions should be used to perform basin level time-series analysis and never be used for analysis at a single grid point”. Furthermore, the way groundwater recharge has been estimated from minimum and maximum storage anomalies while disregarding groundwater discharge will lead to substantial underestimation of groundwater recharge in areas where groundwater interacts with surface water. Variations in hydrological nomenclature are not necessarily problematic per se as long as they are clearly defined. However, redefining recharge in this way means the comparison presented in Supplementary Table 4 in ref. ^3^ with existing global-scale recharge estimates that all use a more hydrogeologically standard definition of recharge (that is, as rates of aquifer replenishment) is effectively ‘comparing apples and oranges’. We are at a loss to see how the authors can claim “High confidence on the globally aggregated […] groundwater volumes”^3^. The paper^3^ states that multiple levels of likelihood are defined for each ESB but we see no rigorous attempt to do so for the groundwater boundary.

This flawed approach to the subglobal ESB is then considered globally to see whether it is met “for all aquifers around the world”^3^. However, this is inappropriately^13^ reported as “sums to 15,800 km^3^ per year global drawdown” (Table 1 in ref. ^3^), which is misleading even when overlooking the definition of ‘drawdown’ as inconsistent with standard groundwater nomenclature. Even if we accepted the definition of the subglobal ESB as being robust, the conclusion that “53% of global land area satisfies ESB”^3^ should not be considered a meaningful statement given the unconstrained uncertainties in the methodology employed. We note a recent study that attempted a global analysis with a different remote-sensing method shows a markedly different spatial pattern of estimated GWS storage trends^14^. It is clear that such analyses are still highly uncertain and more work is needed before they are employed operationally.

In conclusion, we consider the proposed groundwater ESB to be flawed, unsafe and unjust. The conclusion that “53% of global land area satisfies ESB”^3^ should not be considered a meaningful statement. Any useful proposed groundwater ESB needs to be better rooted in robust groundwater theory that accounts for how it relates to the surface water boundary^13^, when and where groundwater is being used, be measurable at the scale that real-world groundwater management is occurring on, and effectively consider how groundwater can be used to reduce environmental injustice. As worthy as the aspiration for this boundary is, the proposed concept and implementation fails in all these crucial respects.

## Data Availability

There are no data associated with this paper.

## Code availability

There are is no code associated with this paper.
